# *Clostridium perfringens* alpha toxin enteritis associated with pulmonary disease in a neotropical otter (*Lontra longicaudis*, Olfers, 1818) under human care

**DOI:** 10.29374/2527-2179.bjvm006724

**Published:** 2024-12-30

**Authors:** Beatriz Araujo dos Santos, Bruna Emely Pereira Barbosa, Arthur Carlos da Trindade Alves, Bernardo de Paula Miranda, Gabrielly Ferreira Santos, Daniel de Almeida Balthazar

**Affiliations:** 1 Undergraduate in Veterinary Medicine, Instituto de Veterinária (IV), Universidade Federal Rural do Rio de Janeiro (UFRRJ). Seropédica, RJ, Brazil.; 2 Veterinarian, Bioparque do Rio, Rio de Janeiro, RJ, Brazil.; 3 Veterinarian, Programa de Pós-Graduação em Medicina Veterinária (PPGMV), Departamento de Medicina e Cirurgia Veterinária (DMCV), IV, UFRRJ. Seropédica, RJ, Brazil.; 4 Veterinarian, DSc. DMCV, IV, UFRRJ. Seropédica, RJ, Brazil.

**Keywords:** clostridium infections, digestive system, enterotoxemia, mustelidae, respiratory tract, clostridioses, sistema digestório, enterotoxemia, mustelidae, trato respiratório

## Abstract

*Clostridium perfringens* alpha toxin (CPA) is an important enterotoxemic pathogen linked to gastrointestinal disorders and previously associated with pulmonary disease in other mammals. A young female neotropical otter presented with lethargy, anorexia and steatorrhea, which developed within 24 hours. Veterinary care was provided under anesthesia, during which dehydration, intestinal hypermotility and pulmonary crackling sounds were identified. Hematological tests revealed normochromic normocytic anemia, and a quantitative RT-PCR assay for *Clostridium perfringens* alpha toxin detected a markedly elevated CPA count (43,789 copies of pathogen DNA/uL) in fecal samples, confirming the diagnosis. An abdominal ultrasound exhibited intestinal gas and mucous fecal contents, with normal wall stratification and evolving peristalsis. Chest X-rays and bronchoscopy revealed diffuse serous secretion associated with an underlying inflammatory process, predominantly affecting the left lung. Treatment included hydroelectrolyte replacement, analgesia, antibiotics, and antiemetics, with close monitoring during the critical stage. The patient improved gradually, with regression of clinical signs and the emergence of innate behaviors, and was discharged to the original enclosure after two weeks, supported by updated test results. In conclusion, this study analyzes and details the veterinary approach, diagnosis, and treatment of an acute infectious enteric condition with pulmonary involvement in a neotropical otter under human care.

## Resumo

*Clostridium perfringens* alpha toxina (CPA) é um importante patógeno enterotoxêmico associado a distúrbios gastrointestinais, com relatos anteriores de afecções pulmonares em outros mamíferos. Uma lontra neotropical fêmea jovem apresentou quadro de apatia, anorexia e esteatorréia, que evoluiu dentro de 24 horas. Durante atendimento veterinário, realizado sob anestesia, constatou-se quadro de desidratação, hipermotilidade intestinal e ruídos pulmonares estertores à ausculta. Exames hematológicos revelaram quadro de anemia normocítica normocrômica, e um RT-PCR quantitativo para *Clostridium perfringens* alpha toxina detectou elevação acentuada na contagem de CPA nas amostras fecais (43.789 cópias de DNA patógeno/µL), confirmando o diagnóstico. À ultrassonografia abdominal observou-se conteúdo intestinal gasoso e fecal mucoso, estratificação parietal normal e peristaltismo evolutivo. Radiografias torácicas e broncoscopia revelaram secreção serosa difusa associada a processo inflamatório, predominantemente no pulmão esquerdo. O tratamento incluiu reposição hidroeletrolítica, analgesia, antibioticoterapia, antieméticos e monitoramento intensivo durante a fase crítica. O paciente evoluiu gradualmente, apresentando regressão dos sinais clínicos e expressão de comportamentos naturais, sendo liberado para o seu recinto de origem após duas semanas, com base em exames atualizados. Em conclusão, este estudo analisa e detalha a abordagem veterinária, diagnóstico e tratamento de uma infecção entérica aguda com acometimento pulmonar em uma lontra neotropical sob cuidados humanos.

## Introduction

The neotropical otter (*Lontra longicaudis*, Olfers, 1818) is a small wild carnivore belonging to the family Mustelidae and the subfamily Lutrinae. It is the only species of its genus occurring in Brazil, and it is found in across biomes except the Caatinga. The neotropical otter inhabits semi-aquatic areas, as its activities take place in a terrestrial environment, such as reproduction and parental care (Cheida et al., 2006; Javorouski & Passerino, 2014; Rheingantz et al., 2011).

Their feeding habits are strategic and opportunistic, favoring slow-moving aquatic animals with limited capacity for escape, in order to capture prey with minimal energy expenditure (Pardini, 1998, Quadros & Monteiro-Filho, 2001; Rheingantz et al., 2012, 2017). The diet of these animals consists mainly of fish and crustaceans, although it may also include other taxa, such as amphibians, mollusks, insects, birds, reptiles, small mammals, and even fruit, depending on the availability of these resources in the environment of origin and the season (Quadros & Monteiro-Filho, 2001; Casariego-Madorell et al., 2008; Carvalho-Junior et al., 2010; Rheingantz et al., 2011).

The difficulty in observing these animals in their natural habitat is due to their elusive behavior, which directly affects the accuracy of the information about their conservation status (Rodrigues et al., 2013). According to the IUCN Red List of Threatened Species™, the conservation status of neotropical otters was designated as Near Threatened (NT) in 2022 (Rheingantz et al., 2022). The conservation of these free-living animals is threatened by several factors, including illegal hunting, habitat fragmentation caused by human activity, toxins bioaccumulation, roadkill, and the prevalence of parasitic and infectious diseases (Rheingantz et al., 2018).

In this context, *Clostridium perfringens* is an important enterotoxemic pathogen. It is a gram-positive, anaerobic bacillus that constitutes the microbiota of animals and is capable of producing potent toxins with specific tissue tropism posing a risk to multiple species. It can also produce heat-resistant spores (Rousselet & Leclerc, 2023). *C. perfringens* has the ability to produce up to 17 different toxins, six of which are identified as "typing toxins", these are: alpha (CPA), beta (CPB), epsilon, iota, enterotoxin, and necrotic enteritis B-like, which are used to classify *C. perfringens* isolates into seven types, labeled A through G (Rousselet & Leclerc, 2023).

In carnivores, CPA is the most frequently detected pathogen, with a high prevalence of gastrointestinal disorders (Rousselet and Leclerc, 2023). Additionally, pulmonary disease has been reported in other mammals, where alpha toxin plays a key role in local and systemic vascular effects in clostridial infections (Bunting et al., 1997; Oda et al., 2012). Therefore, it is proposed that this toxin induces endovascular damage and disrupts the accumulation of polymorphonuclear neutrophils in lung lesions associated with *C. perfringens* septicemia (Bunting et al., 1997).

Considering the aforementioned, this study aims to provide a detailed account of the veterinary approach, diagnosis, and successful treatment of an acute enteric condition induced by *C. perfringens* alpha toxin, accompanied by pulmonary involvement, in a of *L. longicaudis* specimen *under* human care.

## Case report

**Figure 1:**
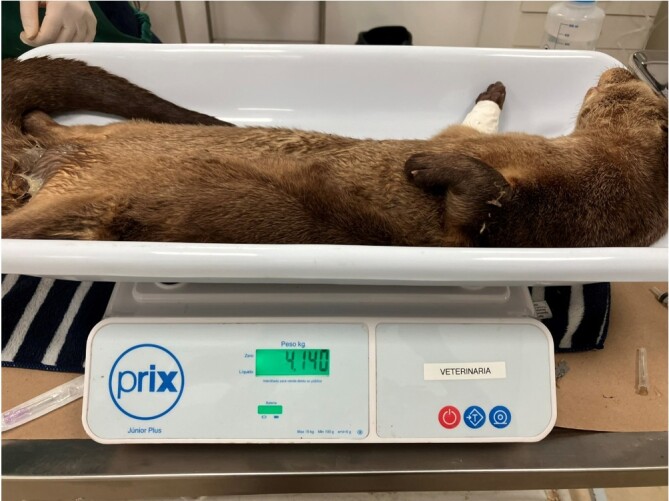
A young female neotropical otter (*Lontra longicaudis*) under sedation, being weighed as part of veterinary care.

**Figure 2:**
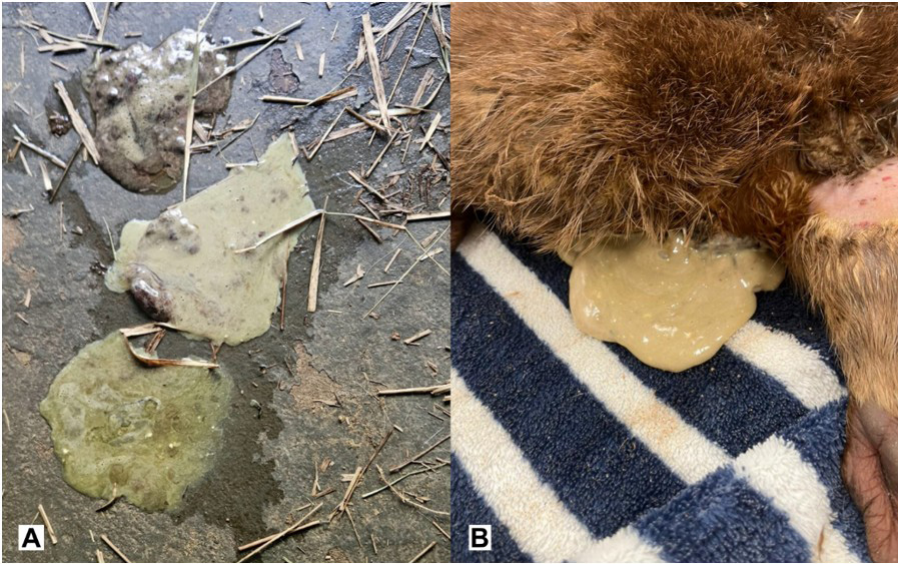
Greyish-yellow liquid feces, indicative of steatorrhea, observed by zookeepers in the enclosure of neotropical otters (*Lontra longicaudis*) (A) and during the physical examination at the veterinary care (B).

A nine-month-old female neotropical otter (*Lontra longicaudis*) weighing 4,14 kg (Figure 1), under human care at a zoo in Rio de Janeiro, Brazil exhibited clinical signs of lethargy, anorexia and steatorrhea (Figure 2), which developed within 24 hours, as observed by the zookeeper. Due to the aggressive natural behavior of the specie, veterinary care was conducted with the animal under chemical restraint. Anesthesia was induced using 6% isoflurane concentration in 100% oxygen in the animal’s transport crate, with effects monitored based on a dose-response observation. The animal was then maintained on 1,5% isoflurane in 100% oxygen administered via a face mask. Subsequently, during physical examination, focal areas of alopecia were identified in the posterior dorsal region, along with 5% dehydration, intestinal hypermobility, and crackling lung sounds through auscultation, correlating with the progression of the previously reported clinical signs.

Biological samples were then collected for complementary tests. Blood analysis revealed a glucose level of 127 mg/dL, a hematocrit of 32% and mean corpuscular hemoglobin concentration (MCHC) at the lower limit, indicative of hypoglycemia and normocytic normochromic anemia, respectively. A coproparasitological examination was carried out using modified Hoffmann, Willis and zinc sulfate flotation techniques, however, no cysts, larvae, or eggs were identified. Additionally, antigen tests for *Giardia* spp. and Parvovirus in the feces were also negative. A quantitative RT-PCR assay for *Clostridium perfringens* alpha toxin detected 43,789 copies of pathogen DNA/uL in fecal samples.

**Figure 3:**
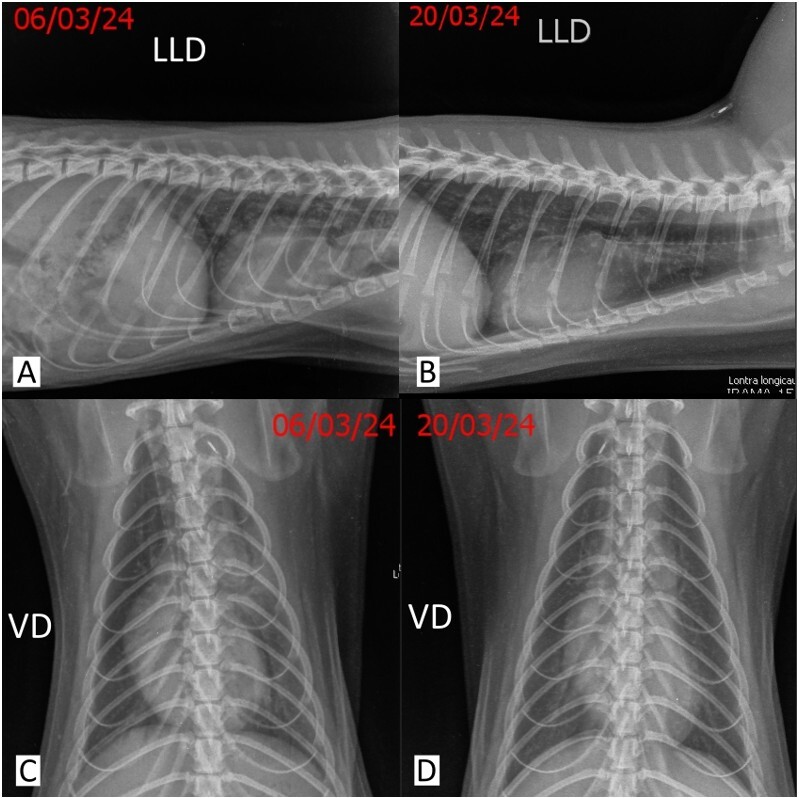
Chest X-rays of a sedated neotropical otter (*Lontra longicaudis*), revealing a diffuse interstitial-alveolar pattern in the cranial and caudal lobes of the left lung during initial care (A and C) and the regression of the changes at the end of treatment (B and D). Right laterolateral (A and B) and ventrodorsal (C and D) views. VD: Ventrodorsal; LLD: Laterolateral direita (Right laterolateral).

The patient was referred for chest X-rays (Figure 3) in ventrodorsal and lateral views, revealing substantial pulmonary involvement characterized by a diffuse interstitial-alveolar pattern in both the cranial and caudal lobes of the left lung. An abdominal ultrasound was also performed, confirming the presence of gas and mucous fecal content within the intestinal loops, with preserved wall stratification and evolving peristalsis.

A catheter was placed in the cephalic vein to initiate the therapeutic protocol, which, during the initial three-day period, comprised fluid therapy with ringer lactate solution (34.2 mL/h, IV), metamizole (25 mg/kg, IM), methadone (0.1 mg/kg, IM), ondansetron (0.2 mg/kg, IV), pentabiotic 6,000,000 UI/mL (0.05 mg/kg, IM), and metronidazole (50 mg/kg, IV). During this period, the animal was sedated daily to maintain the catheter and administer injectable medications. Starting on the third day, when spontaneous feeding resumed, the protocol was adjusted to metamizole (25 mg/kg, PO, BID) and metronidazole (20 mg/kg, PO, BID).

**Figure 4:**
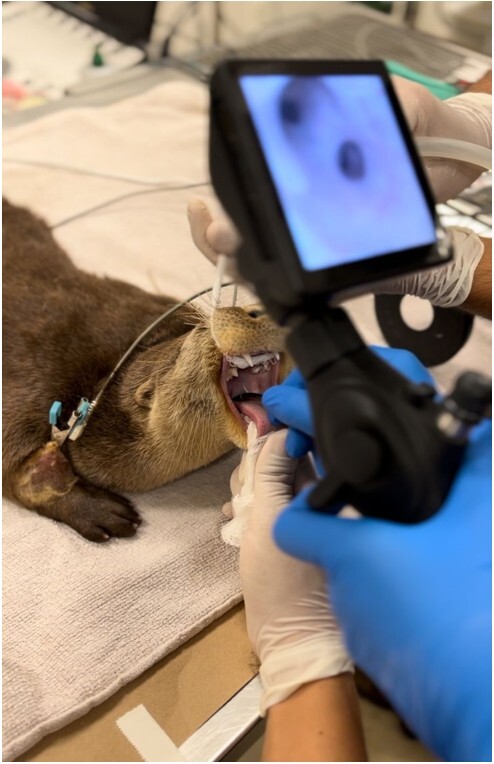
Neotropical otter (*Lontra longicaudis*) under sedation, during the bronchoscopy exam.

**Figure 5:**
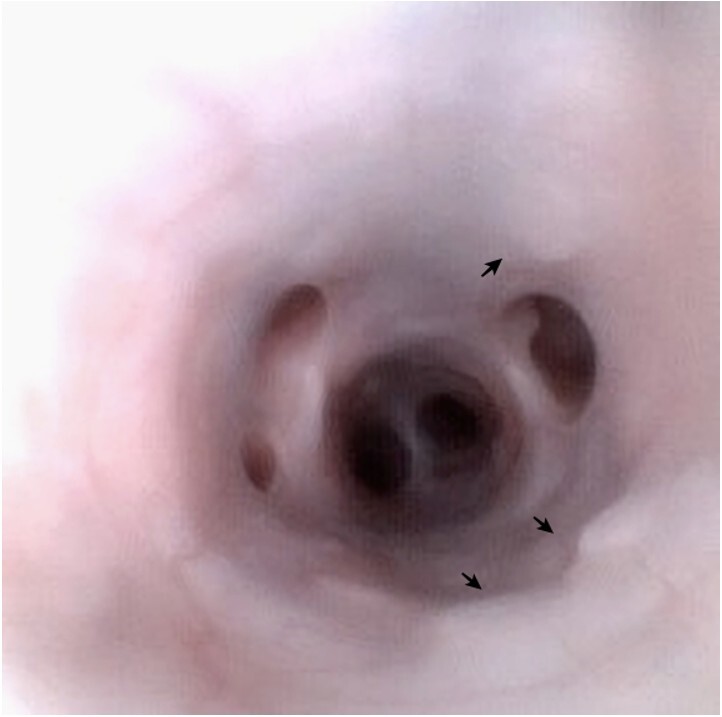
Bronchoscopy of neotropical otter (*Lontra longicaudis*), revealing polypoid areas (arrows) and diffuse serous secretion associated with an inflammatory process in the left caudal lung lobe.

To further elucidate the pulmonary condition, while sedated, the patient underwent a bronchoscopy (Figure 4) after stabilization. The procedure revealed diffuse serous secretion associated with an inflammatory process, predominantly affecting the caudal lobes, with greater intensity in the left lung (Figure 5). During the examination, bronchoalveolar lavage samples were collected, preserved in RNA-Later and sent to the Laboratory of Diversity and Viral Diseases from the Federal University of Rio de Janeiro for detection of SARS-CoV-2 antigen, Influenza A, and Influenza B, as well as for PCR targeting the Coronaviridae family, all of which returned negative results. Cytological analysis was also conducted, using cytocentrifugation, simple smear, and linear concentration methods, revealing a lymphocytic inflammatory process associated with tissue hyperplasia, with no cytological evidence of neoplasia or infection in the sample.

On the next day past the initial treatment, the patient exhibited increased activity and an improvement in stool consistency, which became more formed and less steatorrheic. The animal's clinical condition progressively improved over the subsequent days, with a gradual resolution of clinical signs. By the end of the treatment, the animal exhibited normal behavior, displayed no signs of pain, and maintained food and water intake within normal parameters for its species.

The animal was kept under observation at the veterinary center, and on the 14th day following the initial treatment, it was sedated again for additional follow-up tests. Hematological tests taken on the eighth and fourteenth days indicated an increase of the hematocrit levels, from 49% to 54%, respectively. An updated chest X-ray demonstrated a notable decline in radiopacity in the left cranial and caudal lobes, confirming the regression of the pulmonary condition after the treatment, and the new abdominal ultrasound showed no pathological alterations. Furthermore, the RT-PCR analysis for CPA detected no traces of the pathogen's DNA in the submitted samples. Subsequently, the patient was discharged and transferred back to its enclosure.

## Discussion

The alpha toxin produced by *C. perfringens* i can rapidly induce cellular and tissue damage due to its enzymatic activities, specifically phospholipase C and sphingomyelinase, which lead to the hydrolysis of the phospholipid membrane of cells, including erythrocytes, consequently inducing hemolysis in various species (Nagahama et al., 1996; Rousselet & Leclerc, 2023; Sakurai et al., 2004; Takagishi et al., 2017). Furthermore, an experiment conducted by Takagishi et al. (2017) demonstrated that alpha toxin impedes erythroid differentiation, leading to a reduction in erythroblasts and, consequently, erythrocytes. This results in the development of anemia, which mirrors the initial condition observed in the patient.

In addition to enteritis, the patient exhibited significant respiratory alterations, evident both upon in the clinical examination and imaging. The interstitial-alveolar pattern observed in radiographic images is characterized by the presence of air bronchograms and increased focal unstructured radiopacity, indicative of non-cardiogenic pulmonary edema resulting from fluid exudation from interstitial capillaries (Thrall, 2017). Cytological analysis of the bronchoalveolar lavage sample revealed evidence of inflammatory tissue damage, indicative of generalized inflammatory response. This response was associated with the systemic effects of alpha toxin, which stimulates the synthesis of pro-inflammatory compounds and induces neutrophil accumulation in the vascular endothelium (Bunting et al., 1997; Oda et al., 2012).

Enteritis caused by *C. perfringens* is a true infection, wherein the pathogen establishes itself in the host, suppresses the immune response, multiplies, and produces toxins (Rousselet & Leclerc, 2023). This explains why the treatment involves intravenous administration of broad-spectrum antibiotics, such as metronidazole and penicillin, along with supportive care tailored to the patient's clinical condition and species (Rousselet & Leclerc, 2023; Silva & Lobato, 2015). In this case, t therapeutic success was demonstrated by the regression of clinical signs, notably the improvement in fecal appearance, coupled with favorable chest X-ray findings, and negative results of the RT-PCR analysis for CPA in fecal samples at the conclusion of the treatment period.

Although the origin of the infection in this case was not fully elucidated, the main hypothesis is that it resulted from the delayed ingestion of the food available, following excessive pathogen proliferation. This is possible due to the rapid proliferation of *Clostridium perfringens* across a wide range of temperatures and in anaerobic environments, as well as in aerobic conditions, characteristics that align with those of the otter’s enclosure (Javorouski & Passerino, 2014; Uzal et al., 2016). Following the patient's discharge from the veterinary center, the otters' daily food portions were gradually reduced in quantity and increased in frequency, aiming to reduce food waste, organic matter accumulation in the enclosure, and, consequently, excessive bacterial proliferation in the area. Moreover, portioning the food into smaller amounts reduces the likelihood of excessive consumption, which can lead to slowed intestinal motility and bacterial retention, as well as the absorption of toxins produced by them (Rousselet & Leclerc, 2023).

Additionally, younger animals are more susceptible to enterotoxemia caused by *Clostridium perfringens* compared to older individuals. This increased susceptibility is due to the immature digestive tract of young animals, which has a limited capacity for toxin-inactivating enzymes. This deficiency results in ineffective breakdown of the food bolus passing into the intestine, compromising the efficiency of the digestive process (Prescott, 2013; Rousselet & Leclerc, 2023).

## Conclusion

In conclusion, this report illustrates the pathogenic potential of bacterial infections caused by *Clostridium perfringens* alpha-toxin, which can lead to enterotoxemia and become fatal if not treated promptly and effectively treated. Furthermore, it elucidates the impact of CPA alpha toxin on the respiratory tract, and the findings from complementary tests and imaging examinations. Moreover, it highlights the importance of nutritional and sanitary management for the health of animals under human care, as these are critical control points of zoo-based veterinary medicine. Finally, this case highlights a successful and integrated veterinary approach to disease management, particularly emphasizing the importance of zoos in the conservation of wildlife species such as *L. longicaudis*.
